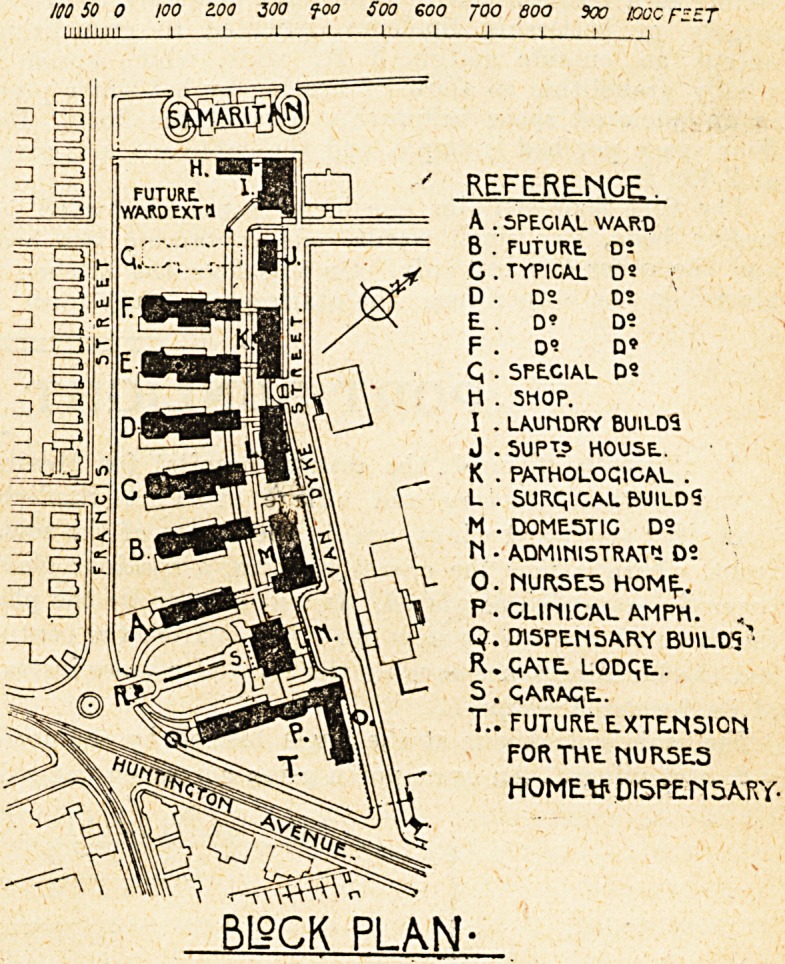# The Peter Bent Hospital, Boston, Mass

**Published:** 1916-11-04

**Authors:** 


### THE PETER BENT HOSPITAL, BOSTON, MASS.

(First Notice.)

This is a general hospital founded through the generosity of Peter Bent Brigham, a native of Vermont and a citizen of Boston, who died in 1877. In accordance with a provision of his will, the hospital was incorporated in 1902. Its buildings were completed and opened in 1913.

To-day we illustrate a block plan and detailed plan of the ground and first floors of a typical pavilion. The positions of these are indicated on the block plan by reference letters c, d, e, and f. Three special wards are shown, being Blocks a, b, and g, the two latter being similar in outline to the typical pavilion illustrated.

A space at the north-west end of the site is available for future ward extension. Towards the eastern end of the site are placed the administration block, and blocks consisting of the nurses' home, clinical amphitheatre, and dispensary. A portion of the site has been reserved for future extension of nurses' home and dispensary. Along the north side of the site, and facing Van Dyke Street, are the following: Domestic buildings, surgical buildings, pathological buildings, superintendent's house, laundry, and shops. These blocks are well placed in that they do not form any obstruction of sunlight to the ward blocks. A main corridor runs practically the whole length of the buildings, off which are short corridors leading to the various blocks.

The distribution of the various buildings is on the whole very satisfactory, wide gaps or open spaces being left between them, and with streets on three sides of the hospital, ample and free circulation of air has been assured. The placing of the wards on the south side of the site, and in positions where a maximum of light and sunshine is obtained, indicates that the welfare of the patients has been the first consideration.

We now deal with the plans of the typical pavilion, and on referring to the ground-floor plan it will be

BLOCK PLAN·

noticed that the staircase, with a lift in the well, is placed immediately off the main corridor and near the entrance to the wards. Close to this is a laboratory, entered from the cross corridor.

PETER BENT BRIGHAM HOSPITAL · BOSTON MASS·

FIRST FLOOR PLAN·

TYPICAL PAVILION·

MAIN OR GROUND FLOOR PLAN·

CODMAN & DESPRADELLE

ARCHITECTS.

31 BEACON ST. BOSTON MASS.

Entering the ward block proper, to the left or east side, are two small wards, one containing two beds and the other one bed. Opposite these are placed a class-room and bathroom with a communicating door, and on the same side are rooms for linen, soiled clothes, and patients' toilet. Leading from a wide space and in a central position is an entrance to the grounds. A large diet kitchen and a duty-room are shown, each having the necessary sinks and fittings. There are two large wards, one, containing eight beds, placed on the east side, and the other, containing eighteen beds, placed at the south end. Before entering the larger ward there is a cross-ventilated corridor running the whole width of the building, and leading off this is a patients' toilet, and next it a bathroom.

It will be noticed that the wards vary considerably in plan, the one at the south end being practically square, and only one storey in height. A large monitor or lantern with glazed sides is indicated, the roof being hipped up to this, and the space included in the ward.

From a photograph sent us the roof appears to be of light iron and wood construction. The rafters, though widely spaced, project below the surface of the sloping roof, thus making it uneven; this, together with the iron-work, must make it difficult to keep clean and free from dust. We do not consider this at all an ideal finish to a ceiling of a hospital ward. The ward floors are composed largely of battleship linoleum cemented to granolithic, except an outside margin 8 ft. wide, which space is wholly granolithic to allow of heating by hot-water pipes in an enclosed space below this part of the floor. The floor space per bed is ample, and the cubic space is given as 2,400 ft.

The first floor contains one large rectangular ward for twelve beds, two small wards, one containing two beds and the other one bed only. On this floor the kitchen is placed in the corner near the staircase; a duty-room is placed centrally, and we are somewhat taken aback to notice that a space for toilet opens from this room. One important point which calls for special comment is the arrangement of the sanitary offices. The general practice of the "cut-off" lobby as applied to English hospitals does not appear to have been favoured, but quite apart from this we do not consider the best arrangement for these offices has been adopted, especially when the patient has to come out of the privacy of the ward and enter them from the corridor as in this case.

A special feature, and one which we welcome, is the ample provision of wide verandahs upon which the patients can be moved.

The hospital covers about ten acres of land, and has accommodation for 225 beds. The construction throughout is fireproof. The single-pipe system of plumbing is used throughout. The architects are Messrs. Goodman and Despadelle, Beacon Street, Boston, Mass.

## Figures and Tables

**Figure f1:**
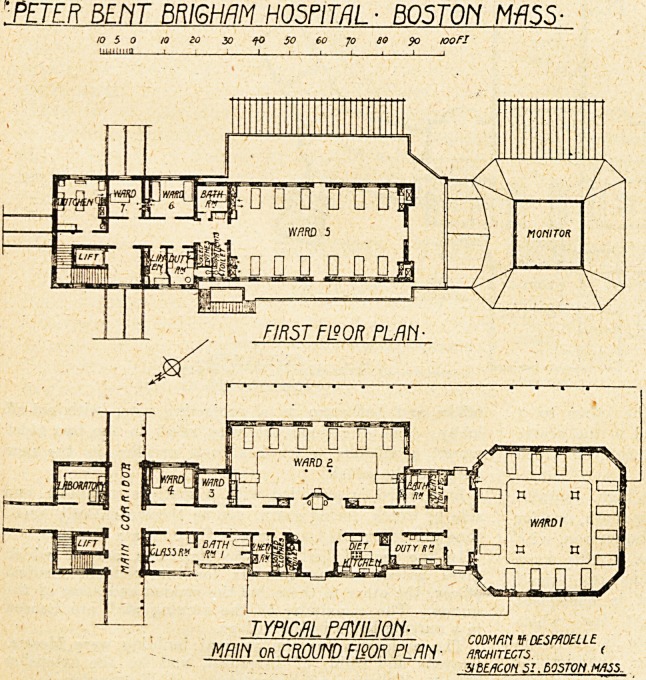


**Figure f2:**